# Overexpression and Tyr421-phosphorylation of cortactin is induced by three-dimensional spheroid culturing and contributes to migration and invasion of pancreatic ductal adenocarcinoma (PDAC) cells

**DOI:** 10.1186/s12935-019-0798-x

**Published:** 2019-03-29

**Authors:** Katharina Stock, Rebekka Borrink, Jan-Henrik Mikesch, Anna Hansmeier, Jan Rehkämper, Marcel Trautmann, Eva Wardelmann, Wolfgang Hartmann, Jan Sperveslage, Konrad Steinestel

**Affiliations:** 1 Gerhard-Domagk-Institute of Pathology, University Hospital Münster, Münster, Germany; 2 Department of Medicine A, University Hospital Münster, Münster, Germany; 3 Division of Translational Pathology, Gerhard-Domagk-Institute of Pathology, University Hospital Münster, Münster, Germany; 4 Institute of Pathology and Molecular Pathology, Bundeswehrkrankenhaus Ulm, Ulm, Germany

**Keywords:** Pancreatic ductal adenocarcinoma, Cortactin, Tumor cell migration and invasion

## Abstract

**Background:**

The nucleation-promoting factor cortactin is expressed and promotes tumor progression and metastasis in various cancers. However, little is known about the biological role of cortactin in the progression of pancreatic ductal adenocarcinoma (PDAC).

**Methods:**

Cortactin and phosphorylated cortactin (Y421) were investigated immunohistochemically in 66 PDAC tumor specimens. To examine the functional role of cortactin in PDAC, we modulated cortactin expression by establishing two cortactin knockout cell lines (Panc-1 and BxPC-3) with CRISPR/Cas9 technique. Cortactin knockout was verified by immunoblotting and immunofluorescence microscopy and functional effects were determined by cell migration and invasion assays. A proteomic screening approach was performed to elucidate potential binding partners of cortactin.

**Results:**

Immunohistochemically, we observed higher cortactin expression and Tyr421-phosphorylation in PDAC metastases compared to primary tumor tissues. In PDAC cell lines Panc-1 and BxPC-3, knockdown of cortactin impaired migration and invasion, while cell proliferation was not affected. Three-dimensional spheroid culturing as a model for collective cell migration enhanced cortactin expression and Tyr421-phosphorylation. The activation of cortactin as well as the migratory capacity of PDAC cells could significantly be reduced by dasatinib, a Src family kinase inhibitor. Finally, we identified gelsolin as a novel protein interaction partner of cortactin in PDAC.

**Conclusion:**

Our data provides evidence that cohesive cell migration induces cortactin expression and phosphorylation as a prerequisite for the gain of an invasive, pro-migratory phenotype in PDAC that can effectively be targeted with dasatinib.

**Electronic supplementary material:**

The online version of this article (10.1186/s12935-019-0798-x) contains supplementary material, which is available to authorized users.

## Background

Pancreatic ductal adenocarcinoma (PDAC) is one of the most common malignancies worldwide, and the fourth leading cause of cancer death in the world [1]. By the time of diagnosis, in as many as 80% of patients the tumor has already metastasized, leading to a 5-year survival rate of less than 5% [2]. In this context, understanding metastatic progression and the highly invasive potential of PDAC cells has become increasingly important. While single-cell invasion and metastatic dissemination upon epithelial-mesenchymal transition had long been held responsible for the spread of tumor cells throughout the body, alternative and more complex models of metastasis have recently been developed. Among those, three-dimensional reconstruction of histological slides as well as in vitro studies have provided evidence for the hypothesis that in fact many epithelial tumors migrate and invade in a collective manner, forming cohesive tumor cell clusters [3, 4].

Irrespective of a single-cell or cohesive cell mode of invasion, many of the steps involved in metastatic progression require the remodeling of the actin cytoskeleton. The altered expression of key regulatory proteins of the actin cytoskeleton, such as cortactin, contribute to tumorigenesis [5]. Initially, cortactin was identified as a major substrate of v-Src kinase, and was named cortactin due to its localization at cortical actin structures [6]. As a scaffolding protein and an actin-related protein 2/3 (Arp2/3) complex activator, cortactin is involved in various cell functions, including actin polymerization and stabilization of F-actin filaments, the formation of focal adhesions and cell motility structures such as podosomes and invadopodia, and extracellular matrix protein deposition [7, 8]. In a pathological context, these functions can lead to deregulated cell migration and invasion [9, 10].

Elevated cortactin expression has been linked to tumorigenesis, and has been shown to correlate with poor clinical outcome in breast cancer [11], head and neck squamous cell carcinoma [12], hepatocellular carcinoma [13], colorectal adenocarcinoma [14], and melanoma [15]. In vitro, cortactin overexpression and activation by Src-mediated tyrosine phosphorylation leads to increased migration of fibroblasts and endothelial cells [16, 17]. Phosphorylation of cortactin occurring primarily at tyrosine 421 (Tyr421) [18], enhances the recruitment of SH2-domain proteins, the activation of Arp2/3 complex and focal adhesion stability and turnover [7, 19, 20]. Tyrosine phosphorylation of cortactin also correlates with invadopodia activity necessary for matrix degradation and cell invasion and is therefore often used as an invadopodial marker [21].

In PDAC, an invasive cancer with high metastatic potential, remodeling of the actin cytoskeleton, changes in cell–cell adhesions, and enhanced migratory potential all might be based on cortactin-mediated actin dynamics. Previously published data suggests an important role for cortactin in PDAC, indicating an association between high cortactin expression and decreased survival time [22]. However, the specific cellular and molecular mechanisms involved in PDAC cell migration and invasion remain unclear. In our study, we analyzed the role of cortactin and its biological function in the tumor progression of PDAC.

## Materials and methods

### Patients, tumor specimens, and tissue microarray

In total, 66 formalin-fixed, paraffin-embedded (FFPE) tissue samples of 39 PDAC patients (14 women, 25 men; median age at diagnosis 65 years, range 39–84 years of age) were included in this study. The tumor specimens were derived from primary tumors and/or metastases (clinicopathological data Table 1). For the PDAC tissue microarray (TMA) construction, two areas within the tumor tissues were selected by two pathologists (K.S. and J.R.). To account for tumor heterogeneity, two tissue cores (diameter 1 mm; tumor/metastasis center) were sampled (132 cores from 66 blocks and 39 patients). Occasional necrobiotic areas and their neighborhood were excluded from TMA sampling to avoid the detection of secondary (e.g. hypoxia-induced) alterations. The study was approved by the Ethics Committee of the University of Münster (Approval No. 2015-102-f-S).

**Table 1:** Clinicopathological data of PDAC patients

Variable	n (%)
Number of patients (%)	39 (100)
*Age*	
Median	65
Range	42–84
≤ 65	22 (56.5)
> 65	17 (43.5)
*Sex*	
Female	14 (35.9)
Male	25 (64.1)
*Type*	
Primary tumor	13 (33.3)
Metastasis	26 (66.6)
*UICC stage*	
I + II	12 (30.8)
III + IV	27 (69.2)
*Grading*	
G2	18 (46.2)
G3	16 (41)
N/A	5 (12.8)

### Immunohistochemistry

Immunohistochemical staining of 4 µm thick sections (deparaffinized in xylene and rehydrated using standard procedures) was performed with a Bench-Mark ULTRA Autostainer (Ventana). After antigen retrieval and pretreatment with citrate buffer, the primary antibodies (cortactin, polyclonal rabbit, 1:50, #LS-B5768, phospho-cortactin, Tyr421, polyclonal rabbit, 1:50, #LS-C353975, LSBio) were incubated (30 min, RT). After washing, the sections were incubated with biotinylated secondary antibodies. Immunoreactions were visualized using the 3-amino-9-ethylcarbazole chromogen as a substrate (Ventana Optiview DAB IHC detection Kit, Ref: 760-700; Roche Diagnostics). Breast cancer tissue was used as a positive control for cortactin/phospho-cortactin (Tyr421) immunostaining, and omitting the primary antibody from the staining protocol served as negative control. Staining was graded as absent, low (weak staining signal and/or immunoreactivity in < 50% of tumor cells) or high (strong staining signal and/or immunoreactivity in ≥ 50% of tumor cells). Scoring was performed independently by two pathologists (K.S. and J.R.).

### Cell lines and reagents

The PDAC cell lines BxPC-3, Capan-1, Capan-2, MIA PaCa-2, Panc-1 and MCF-7 were obtained from Deutsche Sammlung von Mikroorganismen und Zellkulturen (DSMZ). AsPC-1, Panc89 and PT45 were generously donated by Prof. Bence Sipos (Tübingen, Germany). All cell lines were maintained in Roswell Park Memorial Institute medium 1640 (RPMI) or Dulbecco’s Modified Eagles’ medium (DMEM), supplemented with 10% FCS (Life Technologies) under standard cell culture conditions (37 °C, 5% CO_2_) to 70% confluence. For spheroid culture, cells were seeded in six-well plates coated with soft agar. The cells were cultured for 6 days if not indicated otherwise, and the medium was supplemented with FCS every 2 days. Inhibitor treatment was performed with increasing concentrations of dasatinib (Santa Cruz Biotech) and DMSO as vehicle control.

### Generation of cortactin knockout cells

Cortactin knockout (CTTN KO) and control cell lines were generated using human CRISPR/Cas9 Cortactin KO Plasmid and CRISPR/Cas9 Control Plasmid (Santa Cruz Biotech). Panc-1 and BxPC-3 cells were transfected with the CRISPR plasmids using lipofectamine 3000 reagent (Thermo Fisher Scientific) according to the manufacturer’s instructions. Twenty-four hours after transfection, transfected cells (GFP^+^) were isolated from untransfected cells (GFP^−^) by fluorescent activated cell sorting (FACS). Cells were cultured as single-cell clones, and cortactin KO was confirmed using immunoblotting and immunofluorescence analysis.

### Immunoblot analysis

Whole cell protein lysates were extracted using cell lysis buffer (Cell Signaling Technology) following the manufacturer’s instructions and protein concentration was determined using the Bradford Assay Kit (Sigma-Aldrich). Immunoblotting was performed according to standard methods. The following primary antibodies were used according to the manufacturer’s instructions: cortactin (#3502), phospho-cortactin Tyr421 (#4569), ERK1/2 (#4695), phoshpho-ERK1/2 Thr202/Tyr204 (#4370), GAPDH clone D16H11 (#5174), Src (#2109) and phospho-Src Tyr416 (#2101), gelsolin D9W8Y (#12953) (all rabbit host species and obtained from Cell Signaling Technology) and E-cadherin NCH-38 (#MA5-12547, mouse, Thermo Fisher Scientific). After Secondary HRP-labeled antibody (rabbit #1706515, mouse #1706516, both obtained from Bio-Rad Laboratories) incubation (1 h, RT) immunolabeling was detected using an enhanced Chemiluminescence Detection Kit (SignalFire ECL Reagent; Cell Signaling Technology) and the Molecular Imager ChemiDoc system (Image Lab Software; Bio-Rad Laboratories).

### Immunofluorescence analysis

Cells were seeded in two-well chambers (Sarstedt) with 5–8 × 10^4^ cells per well. After a washing step with cold PBS, cells were immediately fixed with cold PFA (4%) for 15 min. After washing, cells were permeabilized with triton X-100 (0.1%) in PBS for 5 min and blocked with 5% BSA in PBS for 30 min. The primary antibodies (cortactin H222 (#3503, Cell Signaling Technology), phospho-cortactin Tyr421 (LSBio) and gelsolin ERP1942 (#ab109014, Abcam)) were incubated at 4 °C overnight. AlexaFlour-488- or AlexaFlour-568-conjugated secondary antibodies (Invitrogen) were incubated for 2 h at RT. Phalloidin (AlexaFlour-568 labeled, Invitrogen) was added for the last 30 min. The slides were mounted with Vectashield mounting medium with DAPI (Vector Laboratories Inc.).

### Proliferation assay

Cell proliferation was monitored in real-time using the xCELLigence RTCA DP instrument (ACEA Biosciences Inc.). Cells were seeded in duplicates or triplicates in 16-well E-view plates (10^4^ cells/well) and monitored for 90 h. Real-time changes in electrical impedance were expressed as “cell index”, a dimensionless parameter representing the cell number.

### Cell migration assay using the xCELLigence system

For migration assays triplicates of 4 × 10^4^ cells were seeded in 130 µl serum-free medium in the upper compartment of the CIM plate of the xCELLigence RTCA DP instrument for real-time cell migration analysis. The lower compartment contained 2.5% FCS to stimulate migration or 0% FCS as a negative control. The cell index representing the amount of migrated cells was calculated using RTCA software from ACEA Biosciences from the change in impedance of the microelectrode sensors when cells adhere to the bottom surface of the microporous membrane. For calculation of the relative cell migration, the cell index after 8 h was normalized to controls and depicted as a bar chart (GraphPad Prism 7, GraphPad Software). Experiments were repeated at least three times. Differences between data points were assessed for statistical significance using two-tailed Student’s t-test comparisons and considered significant at *p* < 0.05.

### Scratch assay

In addition, cell migration was measured by performing scratch assays. Cells were grown to full confluence in growth medium. A scratch was made in the cell monolayer using a sterile pipette tip, and cells were washed twice with PBS and cultured in medium supplemented with 2% FCS for up to 18–24 h. Migration was monitored by taking images at the beginning (0 h) and at the predefined cell line-dependent endpoint (BxPC-3, 18 h; Panc-1, 24 h). The percentage of the closure of the gap was analyzed using NIH ImageJ software. Experiments were repeated at least three times and minimum 12 scratches were recorded per experiment.

### Collagen invasion assay

Cell invasion was analyzed measuring the spreading of spheroids embedded in collagen after 24 h. Prior to seeding BxPC-3 cells as spheroids (5 × 10^3^ cells/spheroid), cells were stained by DiO (Invitrogen) for 20 min at 37 °C and washed twice with PBS. After overnight culturing, each cell spheroid was harvested and embedded in 40 µl of a solution of 1% methyl cellulose in RPMI medium. Spheroids were then mixed with 60 µl of 3 mg/ml collagen matrix (Rat Tail Collagen Type I, Corning) and seeded in 96-well plates. After incubation for 30 min at 37 °C, the gelled collagen was detached from the well wall with a pipette tip to ensure proper media supply, and wells were filled with 100 µl of complete media. Images were taken at the beginning and after 12 and 24 h using an inverted fluorescent microscope (Olympus). The cross-sectional areas of the spheroid and the invasion area were analyzed with the ImageJ software. The percentage of cell invasion was calculated by normalizing the cross-sectional area at 12 and 24 h to that at 0 h. At least three independent experiments were performed (each in quintuplicates).

### Matrix degradation assay

A QCM Gelatin Invadopodia Assay (EMD Millipore) was performed according to the manufacturer’s instructions. Two-well glass chamber slides (Nunc Lab Tek II, Thermo Fisher) were coated with Cy3 (red) gelatin. 5 × 10^4^ cells in RPMI 1640 supplemented with 10% FCS were seeded onto the fluorescent gelatin matrix and incubated for 24 h. Cells were then fixed and stained with phalloidin. Matrix degradation was quantified as the proportion of cells associated with the degraded gelatin matrix, as defined by the formation of at least one degradation patch underneath the cell, regardless of its size, and the total number of cells counted. Experiments were repeated three times and data in 10 fields at a magnification of 20× were used for the calculation.

### Immunoprecipitation

Immunoprecipitations (IP) were performed using the µMACS Protein A/G MicroBeads Kit (Miltenyi Biotec) according to the manufacturer’s instructions. Whole cell lysate of 5 × 10^6^ cells was mixed with cortactin 4F11 mouse monoclonal antibody (EMD Millipore; 1 µg per 500 µg protein lysate) and 50 µl Protein G MicroBeads, and incubated for 1 h on ice. For magnetic IP, the cell lysate was passed over a separation column placed in the magnetic field of a µMACS separator and washed four times with 200 µl cell lysis buffer. To conduct SDS-PAGE and immunoblotting analysis, the immune complex was eluted from the column using heated 1× SDS sample buffer. Rabbit antibodies (see Immunoblot analysis protocol) were used for immunoblotting to avoid the detection of any interfering IgG bands from the mouse antibody used during the IP protocol. Protein–protein interactions were depicted by immunoblotting, using antibodies specific to the putative co-immunoprecipitated targets. For mass spectrometric analysis, aliquots of the IP eluates were analyzed by Coomassie blue staining (Brilliant Blue R Concentrate; #B8647; Sigma) following SDS-PAGE (40 µl). Isolated bands were subjected to mass spectrometry (Core Unit Proteomics, Interdisciplinary Center for Clinical Research University of Münster, Germany).

## Results

### Enhanced cortactin expression level and phosphorylation correlates with tumor metastasis

**Fig. 1:**
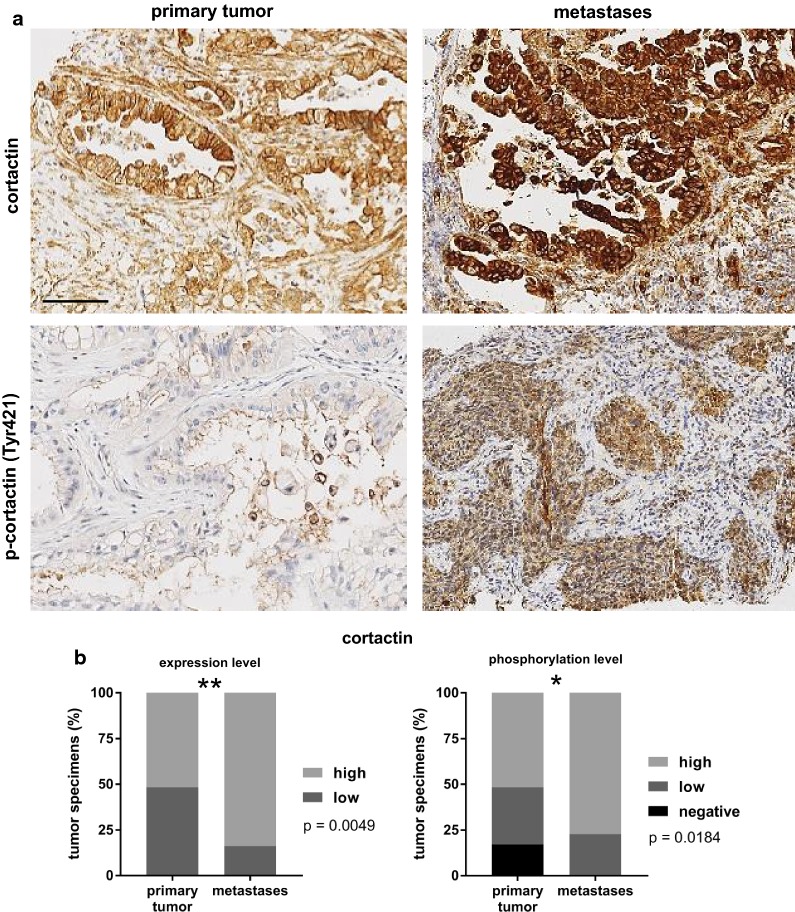
High expression and phosphorylation of cortactin is associated with metastatic manifestation of pancreatic cancer cells. **a** Immunohistochemical staining of representative cases of low and high cortactin and phospho-cortactin (Tyr421) expression in microarray tissues. Scale bar, 100 µm. **b** Immunohistochemical stainings of tumor tissues are summarized as bar charts indicating the percentage of cases with a negative, low or high staining intensity

The expression and the activation status of cortactin was determined by immunohistochemical staining of PDAC specimens. Cortactin was expressed in all tissue samples. However, PDAC metastases revealed a significant upregulation of cortactin (p = 0.0049 Chi square) and phospho-cortactin (p = 0.0184 Chi square) compared to those from primary tumors (Fig. 1a + b). More specifically, immunoreactivity was low in 48.3% (14 of 29 samples) and high in 51.7% (15/29) of primary tumor tissues. In metastatic tissue cores, however, cortactin expression was low in 16.2% (6/37) and high in 83.8% (31/37) cases (Fig. 1b). Tyr421 phosphorylated cortactin was detected in 92% of 64 tumor cores (two of the 66 tissue samples were excluded from further analysis due to low tumor percentage (< 5%); 8% of the cases showed negative tumor cells. Phospho-cortactin staining intensity was absent/negative in 5 (17.2%), low in 9 (31.0%) and high in 15 (51.8%) of primary tumors, while staining was negative in 0, low in 8 (22.9%) and high in 27 (77.1%) metastatic tumors. There were no differences in cortactin expression levels according to other clinic-pathological variables (age, sex, grade of tumor). No significant correlations were observed for the overall survival rate, but patients with high cortactin expression had a trend towards shorter overall survival, as illustrated in Additional file 1: Figure S1.

In cryo-conserved PDAC samples of matched non-neoplastic pancreatic and tumor tissue of 11 patients analyzed via qRT-PCR (Additional file 2: Materials and methods) no significant dysregulation of cortactin at the mRNA level was observed (Additional file 1: Figure S2).

### Cortactin expression and cortactin KO in PDAC cell lines

**Fig. 2:**
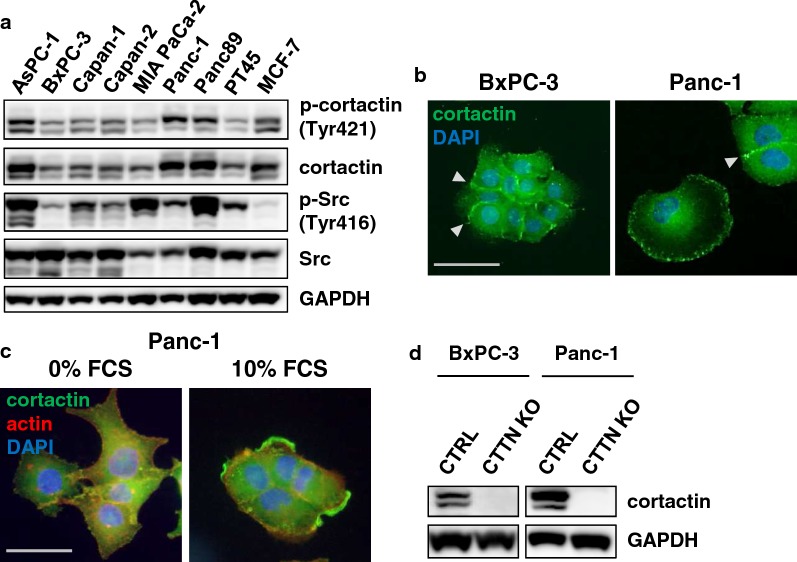
Characterization of cortactin expression and subcellular localization in PDAC cell lines and establishment of cortactin knockdown mediated by CRISPR/Cas9 technology. **a** Immunoblotting of whole cell lysates of PDAC cell lines for cortactin and Src and their activated forms (p-cortactin Tyr421 and p-Src Tyr416). The MCF-7 breast carcinoma cell line was included as positive control. **b** Cortactin localization (green) in BxPC-3 and Panc-1. The nucleus was counterstained with DAPI (blue). Arrowheads indicate cortactin expression at sites of cell–cell-contacts. Scale bar, 50 µm. **c** Serum starved Panc-1 cells were stimulated with 10% FCS for 1 h. After stimulation, cortactin (green) localized to the cell membrane. The actin cytoskeleton was stained with phalloidin (red) and the nucleus was counterstained with DAPI (blue). Scale bar, 50 µm. **d** Immunoblotting of CRISPR/Cas9 mediated cortactin knockdown (CTTN KO) cells. Cells were grown as single selected clones. GAPDH was used as loading control

Immunoblotting of whole protein cell lysates of eight PDAC cell lines and the breast carcinoma cell line MCF-7 as a positive control [23] showed cortactin expression in all tested cell lines (Fig. 2a). Furthermore, Panc-1 cells demonstrated the highest amount of Tyr421-phosphorylated cortactin. There was no correlation between the phosphorylation levels of cortactin and the cortactin regulator Src (Fig. 2a). The subcellular localization of cortactin was analyzed in BxPC-3 and Panc-1 cells using immunofluorescence microscopy. Cortactin is expressed at cell–cell contacts (grey arrows), as well as in the lamellipodia of the cells (Fig. 2b). Previous research has demonstrated that cortactin localizes to the cell membrane after stimulation with growth factors and promotes actin polymerization and cell migration [15]. To investigate whether cortactin is functionally active in PDAC cell lines, cells were serum-starved for 24 h and then stimulated with 10% FCS for 1 h. Immunofluorescence staining for cortactin and phalloidin to visualize the actin cytoskeleton showed redistribution of cortactin to the membrane ruffles in Panc-1 cells after serum stimulation (Fig. 2c).

To further assess the functional role of cortactin in PDAC cell lines, we applied CRISPR/Cas9 gene editing for depletion of cortactin (CTTN KO) in BxPC-3 and Panc-1 cell lines. Immunoblots of BxPC-3 and Panc-1 CTTN KO and control cells are shown in Fig. 2d. In immunofluorescence, CTTN KO cells showed a slight, collapsed fluorescent signal around the nucleus. However, no signal was observed at the cell periphery or cell–cell contacts (Additional file 1: Figure S3), indicating that cortactin was functionally inactive.

### Knockout of cortactin decreases cell motility

**Fig. 3:**
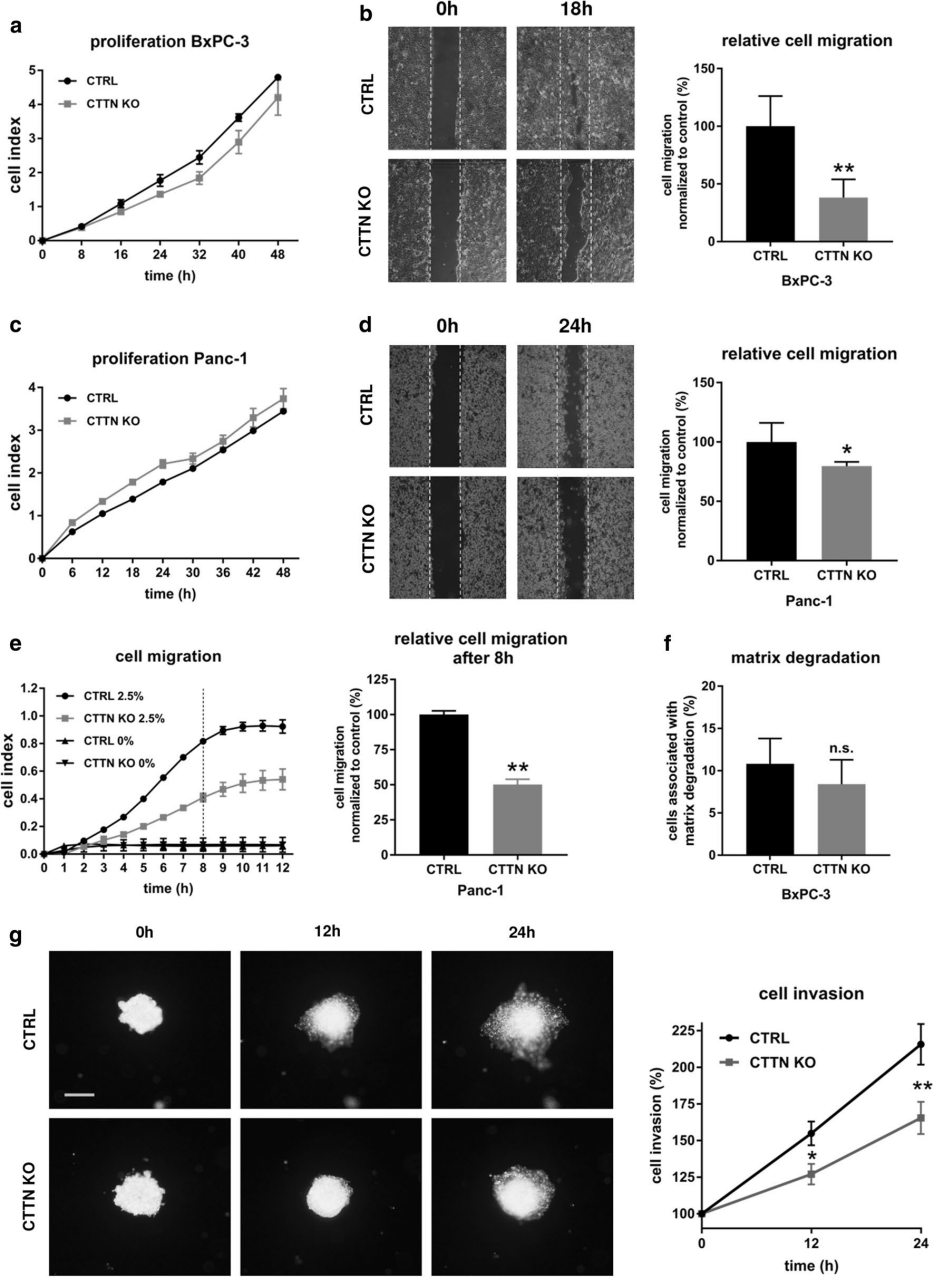
Functional analysis of cortactin knockdown (CTTN KO) cells and controls (CTRL). **a**, **c** Knockdown of cortactin did not affect pancreatic cell proliferation measured with the xCELLigence system of BxPC-3 and Panc-1 cells. **b**, **d** Cell migration assessed in vitro performing scratch assays showed significantly impaired migration of CTTN KO cells compared to the controls. **e** Cell migration stimulated with 0–2.5% FCS was measured with the xCELLigence system and showed significantly reduced migration in Panc-1 CTTN KO cells. Bar chart indicates the relative cell migration normalized to control cells after 8 h. **f** Quantification of matrix degradation. No significant differences were observed between BxPC-3 CTTN KO cells and controls. **g** BxPC-3 cells were stained with DiO prior to spheroid culture for collagen cell invasion assays. Spheroids cultured for 24 h were then embedded in a collagen suspension and cell invasion was assessed 12 and 24 h after plating. BxPC-3 CTTN KO cells showed significantly impaired invasion compared to the controls. Scale bar, 1000 µm. *p < 0.05; **p < 0.01; ***p < 0.001; ns, not significant. Data points shown are the mean + SD. The results are representative of at least three independent experiments

In proliferation assays using the xCELLigence system no differences between BxPC-3 and Panc-1 CTTN KO and control cells were observed (Fig. 3a, c).

Scratch assays performed with BxPC-3 and Panc-1 CTTN KO cells showed a significant decrease (30–50%) in migratory capacity (Fig. 3b, d) compared to controls. Cell migration measured with the xCELLigence technique showed a significant reduction in cell migration of up to 50% in Panc-1 CTTN KO cells compared to controls (Fig. 3e). Since cortactin is also associated with matrix-metalloproteinase secretion [24, 25], we performed gelatin degradation assays. Here, a slight but not significant tendency towards diminished matrix degradation potential was observed after cortactin KO (Fig. 3f). In collagen invasion assays BxPC-3 CTTN KO cells showed significantly impaired cell invasion into the collagen matrix compared to control cells (Fig. 3g). Together, these results indicate a pivotal role for cortactin in PDAC cell invasion in vitro.

### Cortactin levels are enhanced after three-dimensional cell culturing and suppressed by dasatinib

**Fig. 4:**
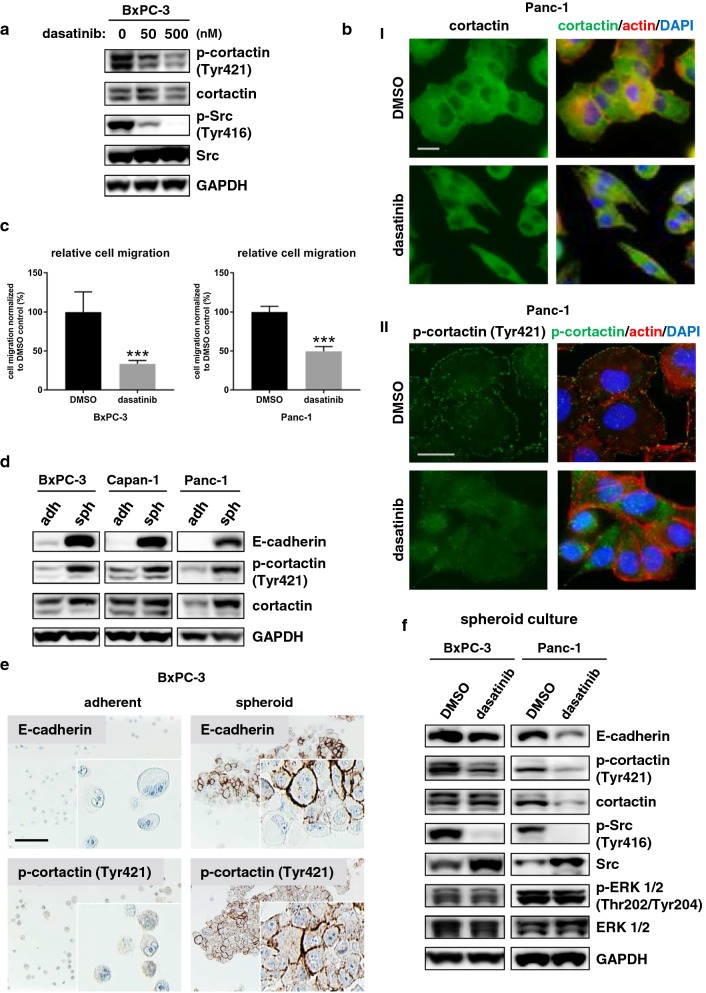
Src inhibitor dasatinib mediated effects in pancreatic cancer cell lines adherently cultured or as spheroids. **a** Dasatinib treated adherently cultured BxPC-3 cells showed decreased Src Tyr416 and cortactin Tyr421-phosphorylation levels compared to control cells. **b** Immunofluorescence analysis of (I) cortactin or (II) p-cortactin Tyr421 after dasatinib treatment. Green fluorescence indicates (I) cortactin or (II) p-cortactin staining, the actin cytoskeleton is shown in *red* and the nucleus is shown in blue. **b I** Morphological changes (epithelial to mesenchymal) after dasatinib treatment (1 µM) were detected in Panc-1 cells. Scale bar, 20 µm. **b II** Immunofluorescence for *p*-cortactin Tyr421 revealed a delocalization of phosphorylated cortactin from the cell membrane to the cytoplasm after dasatinib treatment. Scale bar, 20 µm. **c** In scratch assays, BxPC-3 and Panc-1 cells treated with 50 nM dasatinib or DMSO as control showed a significant decrease in cell migration ***p < 0.001. **d** Increased cortactin expression and Tyr421 phosphorylation levels after 6 days of spheroid culture compared to adherently cultured cells were detected in pancreatic cancer cell lines BxPC-3, Capan-1 and Panc-1. adh, adherent; sph, spheroid. **e** Elevated Tyr421-phosphorylation of cortactin and higher expression of E-cadherin at cell–cell contacts of agarose embedded BxPC-3 cells in spheroid cultured cells (6 days) were determined by immunohistochemistry. Scale bar, 100 µm. **f** Immunoblotting of BxPC-3 and Panc-1 cells cultured in spheroids and treated with dasatinib (BxPC-3: 100 nM; Panc-1: 1 µM) for 24 h showed less induction of E-cadherin and cortactin expression compared to control cells. Immunoblotting of p-ERK 1/2 (Thr202/Tyr204) and ERK kinase were included to verify dasatinib specificity. GAPDH was used as loading control

Since cortactin is a target of Src family kinases, we analyzed the role of cortactin in PDAC cell migration by using dasatinib, a Src family kinase inhibitor [26]. In line with previous reports [27], cell viability of Panc-1 cells was resistant to dasatinib (Additional file 1: Figure S4), while dasatinib-sensitive BxPC-3 cells showed decreased cell viability after dasatinib treatment (Additional file 1: Figure S5). After treating BxPC-3 cells with increasing concentrations of dasatinib, cortactin Tyr421-phosphorylation decreased (Fig. 4a). In Panc-1 cells, no effects on cortactin phosphorylation could be detected by immunoblotting, while Src kinase was hypophosphorylated as expected (Additional file 1: Figure S6). Immunofluorescence microscopy revealed a morphological change in Panc-1 cells after dasatinib treatment (Fig. 4b), which was not observed in BxPC-3 cells (data not shown). Upon treatment, Panc-1 cells switched from an epithelial to a spindle cell-like phenotype with a diminished cortactin staining signal at the cell membrane (Fig. 4bI). Although we were unable to detect changes in cortactin activation by immunoblotting in Panc-1 cells (Additional file 1: Figure S6), immunofluorescence analysis revealed a diminishment of phosphorylated cortactin at the cell membrane (Fig. 4bII). However, treating Panc-1 and BxP-3 cells with dasatinib significantly decreased cell migration (Fig. 4c).

The expression of cortactin at cell–cell contacts (Fig. 2b), the morphological switch and diminished expression of phosphorylated cortactin at the cell membrane upon dasatinib treatment (Fig. 4b), and the observation that cortactin phosphorylation is enhanced with increasing cell confluency (Additional file 1: Figure S7), prompted us to investigate if cortactin expression and/or phosphorylation was inducible by culturing the cells on soft agar enabling the formation of three-dimensional cell–cell contacts. Immunoblot analysis of three-dimensionally versus adherently cultured cells revealed increased expression or phosphorylation of cortactin after 6 days in BxPC-3, Capan-1 and Panc-1 cells (Fig. 4d). E-cadherin, a cell–cell contact molecule that has been shown to be upregulated or re-expressed in hepatocytes after spheroid formation [28], was also elevated in all three cell lines when they were grown as spheroids.

Immunohistochemical staining of agarose-embedded BxPC-3 cells confirmed the high expression of phosphorylated cortactin and E-cadherin at cell–cell contacts when cells were cultured as spheroids (Fig. 4e). Treating spheroid-cultured cells with dasatinib showed a decrease in the spheroids’ stability (data not shown), and immunoblotting of spheroid-cultured BxPC-3 and Panc-1 cells showed reduced E-cadherin and cortactin expression as compared to control cells (Fig. 4f).

### Gelsolin is a possible interaction partner of cortactin

**Fig. 5:**
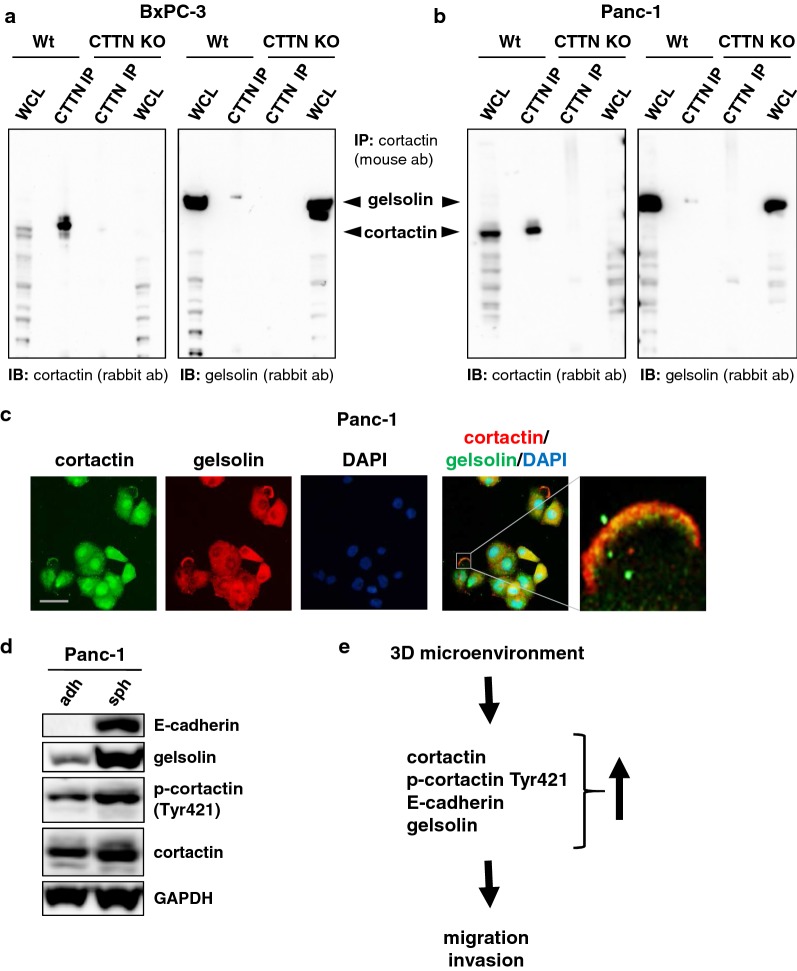
Gelsolin is a novel interaction partner of cortactin in PDAC. **a**, **b** Verification of mass spectrometry results by Co-immunoprecipitation (Co-IP) of gelsolin by cortactin antibody. Co-IP with cortactin antibody showed cortactin precipitation and co-precipitation of gelsolin in **a** BxPC-3 and **b** Panc-1 cells. CTTN KO cells served as negative controls. Black arrows indicate size of gelsolin and cortactin. **c** Immunofluorescence staining of gelsolin (green) and cortactin (red*)* confirmed the possible interaction as co-localization of both proteins could be detected. Scale bar, 50 µm. **d** In cells cultured in spheroids upregulation of gelsolin expression compared to cells in adherent culture was detected. GAPDH was used as loading control. **e** Scheme of the role of cortactin in pancreatic cancer migration. ab, antibody; adh, adherent; IB, immunoblot; IP, Immunoprecipitation; sph, spheroid; WCL, whole cell lysate; Wt, wild type

In a Co-IP approach, followed by mass spectrometry in addition to known cortactin binding partners such as Arp2/3, gelsolin was identified as a protein associated with cortactin in PDAC. Further Co-IP experiments and immunoblotting confirmed interaction of gelsolin with cortactin in Panc-1 and BxPC-3 cells (Fig. 5a + b). Additional immunofluorescence studies revealed co-localization of both proteins at the cell periphery (Fig. 5c).  Along with E-Cadherin and cortactin, Gelsolin was induced during three-dimensional cell culturing of Panc-1 cells compared to adherently cultured cells (Fig. 5d).

## Discussion

PDAC is one of the most aggressive human neoplasms, marked by extensive local (perineural) spread, and early metastasis to lymph nodes or the liver. Due to late symptoms and challenging tumor imaging, most cases are either too advanced for surgery, and/or the tumor has already formed metastases. The presence of metastases is an adverse prognostic factor for PDAC patients [2]. Even after radical surgery, there is a high level of tumor recurrence. Therefore, an understanding of the cellular and molecular mechanisms underlying PDAC cell migration and invasion is crucial for the development of better therapeutic modalities for PDAC patients.

In various cancers, cortactin overexpression is associated with a dismal prognosis and increased metastasis [11–15]. At a mechanistic level, cortactin overexpression may promote invasion and metastasis by facilitating the formation of invasive structures such as lamellipodia and invadopodia, as cortactin has been shown to be required for the formation and proper function of those protrusive structures formed by migrating and invading cells [24, 29]. However, little is known about the biological function of cortactin and its role in cellular and molecular mechanisms involved in the tumor progression of PDAC. In our cohort of tumor specimens from patients with histologically confirmed PDAC, we observed a significant upregulation of cortactin and phosphorylated (i.e. activated) cortactin in metastases of PDAC patients as compared to primary tumors. Patients with low cortactin expression in the primary tumor appeared to have a survival advantage in the first 2 to 3 years after diagnosis (Additional file 1: Figure S1), which is in line with a study by Tsai et al., who demonstrated that high cortactin expression in tumorous tissue significantly correlates with a lower survival rate in a cohort of 50 PDAC patients [22]. However, the correlation of cortactin expression levels with overall survival rates was of no statistical significance in our study, which may be due to the size of the group of patients with low cortactin expression and without metastases. In contrast to primary breast cancers and head and neck squamous cell carcinoma cell lines, for which a correlation of cortactin gene amplification with overexpression at the mRNA level was reported [11, 23, 30], we were unable to detect a significant upregulation of cortactin at the mRNA level when comparing cortactin mRNA expression in fresh frozen samples from PDAC tumor tissue and non-neoplastic pancreatic tissue. This indicates that cortactin in PDAC might either be regulated by post-transcriptional factors, or that the phosphorylation/activation status in addition to the expression level of cortactin might be crucial for its biological relevance in PDAC. Other mechanisms involved in elevated cortactin expression in PDAC might be based on post-transcriptional modification enhancing protein stability. It has been shown that ERK-induced serine phosphorylation of cortactin leads to polyubiquitination and degradation within the proteasome [31]. Deregulated proteolysis might increase cortactin protein stability in cancer with high cortactin expression but without gene amplification.

Since cortactin is phosphorylated by oncogenes (e.g. Src [17], ERK [32], Arg [33]), which are known to be upregulated in PDAC [34, 35], we examined the activation of cortactin via phosphorylation of Tyr421 in our cohort. Cortactin showed significantly greater phosphorylation in metastases compared to primary tumors. Elevated cortactin activation by phosphorylation has been revealed to promote cancer progression in breast cancer and malignant melanoma by, e.g. enhancing the interaction of tumor cells with endothelial cells and elevating the tumor cell’s capacity for cell invasion into tissues [36, 37]. Using cortactin mutants, Li et al. reported that cortactin phosphorylation promoted metastasis in breast cancer cells; cortactin that was deficient in tyrosine phosphorylation induced more than 70% fewer bone metastases than wild type cortactin-expressing cells [36]. Interestingly, it has also been reported that hyperphosphorylated cortactin might suppress cell migration by a negative feedback mechanism, as knockdown of cortactin in gastric cancer cells with high cortactin phosphorylation levels and high migratory potential further enhanced cell migration [38].

In accordance with previous reports [15], in our experiments stimulation with growth factors showed re-localization of cortactin to lamellipodia, indicating functional activity of cortactin in PDAC cell lines. In functional migration and invasion assays as well as proliferation assays with the two PDAC cell lines BxPC-3 and Panc-1, we were able to demonstrate that a CRISPR/Cas9-mediated cortactin knockout resulted in significant reduction in migration and invasion, while proliferation of the PDAC cells was unaltered. It has to be pointed out that, employing of a serum gradient, the xCELLigence assay is unable to clearly distinguish between chemotaxis and migration; however, the effects of CTTN knockout were also observed in scratch assays in the absence of a serum gradient. Comparable results using RNAi knockdown of cortactin were demonstrated by Miyazawa et al., showing that suppression of cortactin decreased CDCP1-PKCδ-mediated cell migration and invasion of BxPC-3 cells [39]. In epithelial cells and fibroblasts, overexpression of cortactin resulted in more motile and invasive cells, while none of the cell types showed altered growth rates or morphologies due to cortactin modulation [16, 21]. Therefore, our results are in line with the well-established role of cortactin in cell motility.

Activated cortactin accumulates at the cell periphery [18]. In three-dimensional cell cultures, which have been shown to reflect an in vivo situation more accurately than monolayer cultures [3] as they allow cells to interact with surrounding cells and to form multi-dimensional structures, cortactin was enhanced and/or hyperphosphorylated. Immunohistochemical staining confirmed the accumulation of phosphorylated cortactin at sites of cell–cell contacts together with the cell–cell contact molecule E-cadherin, which was re-expressed/upregulated in cells cultured in spheroids. E-cadherin has been demonstrated to be important for cell–cell attachment and spheroid formation [28]. In spheroid-cultures as well as in adherent cell cultures, we were able to demonstrate that cortactin activation as assessed with regard to its localization and/or phosphorylation on Tyr421 is pharmacologically targetable by dasatinib, an inhibitor that targets Src family kinases [26]. After treatment, phosphorylation of cortactin decreased and cortactin delocalized from the cell membrane to the cytoplasm. Additionally, dasatinib treatment led to decreased cell–cell contacts and decreased E-cadherin expression in three-dimensional spheroid culture (data not shown). Interestingly, other researchers have observed an increased stability of E-cadherin expression after dasatinib treatment with enhanced integrity of cell–cell adhesions [40, 41], while in our experiments treatment with dasatinib reversed the effects induced by three-dimensional cell culturing. It is therefore well conceivable that three-dimensional cell growth induces cortactin expression and Tyr421-phosphorylation, contributing to a pro-metastatic, migratory phenotype in PDAC.

It has been shown that the modulation of molecular mechanisms in which cortactin is involved, such as F-actin cross-linking and cofilin-cortactin interaction, is pH-dependent [42, 43]. Tumor cells growing in a three-dimensional spheroid may develop an acidic microenvironment when compared to two-dimensional tumor models which might contribute to the phosphorylation of cortactin in vitro as well as in the in vivo situation.

Consistent with the results from others, treatment with the Src family kinase inhibitor dasatinib reduced cell migration in PDAC cell lines [27]. It is possible that the dephosphorylation of cortactin as well as its relocalization that we observed after dasatinib treatment contribute to the inhibitory effect of dasatinib on cell migration. However, the precise mechanism by which cortactin could be utilized to target cancer metastasis must be further investigated. MacGrath and Koleske have provided an excellent review of cortactin as a promising target molecule in cancer therapy approaches [10], showing the importance of clarifying the role of cortactin in PDAC progression as well as of identifying and understanding its interactions with other proteins that contribute to cancer migration and invasion in an effort to combine various strategies in multimodal therapy approaches and thereby prolong recurrence-free survival of PDAC patients.

In this study, we performed co-immunoprecipitation assays to identify novel proteins interacting with cortactin in PDAC, and detected gelsolin as a cortactin-binding partner using mass spectrometry. This finding was confirmed by further co-immunoprecipitation experiments from Panc-1 and BxPC-3 PDAC cell lysates followed by immunofluorescence showing a co-localization of cortactin and gelsolin at the cell periphery. The actin binding protein gelsolin regulates actin dynamics in a Ca^2+^-dependent manner by severing and capping actin filaments and has been shown to modulate several signaling pathways, altering cytoskeleton architecture and cell motility [44]. In an in vitro approach using RNAi, Thompson et al. reported that a reduction of gelsolin significantly impaired pancreatic cancer cell motility [45]. Tanaka et al. described an EMT switch after gelsolin knockdown by siRNA with a conversion of E-cadherin to N-cadherin in mammary epithelial cells [46]. Here, in cells cultured as spheroids with three-dimensional cell–cell contacts, we observed an elevated expression of gelsolin along with E-cadherin re-expression and cortactin induction. Further experiments will be necessary to analyze the functional role of the interaction between cortactin and gelsolin in tumor progression of PDAC.

It has to be stated that low or loss of E-cadherin expression has been postulated to induce EMT and to be a prerequisite for metastatic disease progression [47]. However, carcinomas have shown to be remarkably heterogeneous and to be able to adopt some mesenchymal features while retaining characteristics of well-differentiated epithelial cells like E-cadherin expression [48]. Moreover, most experimental data on cancer cell migration and invasion are derived from two-dimensional cell culture models, while it has been shown that in the in vivo situation, epithelial tumors have the ability to migrate and invade collectively in three-dimensional cell clusters [49]. Data from Gagliano et al. revealed an upregulation of N-cadherin, vimentin and podoplanin in three-dimensionally cultured PDAC cells compared to adherent cell culture [50]. PDAC cells cultured as spheroids or during collective cell migration might therefore gain a ‘partial EMT’ status with upregulated E-cadherin, N-cadherin and vimentin. Cortactin expression and Tyr421-phosphorylation would then contribute to a pro-metastatic, migratory phenotype in PDAC.

## Conclusions

In conclusion, our results identify cortactin and its phosphorylation on Tyr421 as an important molecular player in PDAC progression. Our data provide evidence that upregulation and/or activation of cortactin in a three-dimensional tumor microenvironment might promote migration and invasion in this fatal disease. A short graphical summary of a proposed model based on our results is depicted in Fig. 5e. This may provide an opportunity for cancer treatment by inhibition of kinases that regulate cortactin or by disruption of protein interactions with the SH3 domain of cortactin. The identification of novel binding partners of cortactin may further elucidate the mechanisms how cortactin contributes to the aggressiveness of PDAC cells.

## Additional files

**Additional file 1.** Additional figures.

**Additional file 2.** Additional Materials and methods.

## Abbreviations

Arp2/3: actin-related protein 2/3
BSA: Bovine serum albumin
CTTN: cortactin
DiO: dioctadecyloxacarbocyanine Perchlorate
DMEM: Dulbecco’s Modified Eagles’ medium
DMSO: dimethyl sulfoxide
DSMZ: Deutsche Sammlung von Mikroorganismen und Zellkulturen
EMT: epithelial–mesenchymal transition
FACS: fluorescent activated cell sorting
FCS: fetal calf serum
FFPE: formalin-fixed, paraffin-embedded
GFP: green fluorescent protein
IgG: immunoglobulin G
IP: immunoprecipitations
KO: knockout
mRNA: messenger *RNA*
PBS: phosphate buffered saline
PDAC: pancreatic ductal adenocarcinoma
PFA: paraformaldehyde
PAGE: polyacrylamide gel electrophoresis
RNA: ribonucleic acid
RNAi: RNA interference
RPMI: Roswell Park Memorial Institute medium 1640
RT: room temperature
SDS: sodium dodecyl sulphate
SH3: Src homology 3
siRNA: small interfering RNA
Thr: threonine
TMA: tissue microarray
Tyr: tyrosine

## References

[1] Zhang Q, Zeng L, Chen Y, Lian G, Qian C, Chen S (2016). Pancreatic cancer epidemiology, detection, and management. Gastroenterol Res Pract.

[2] Hidalgo M (2010). Pancreatic cancer. N Engl J Med.

[3] Bronsert P, Enderle-Ammour K, Bader M, Timme S, Kuehs M, Csanadi A (2014). Cancer cell invasion and EMT marker expression: a three-dimensional study of the human cancer-host interface. J Pathol.

[4] Mayor R, Etienne-Manneville S (2016). The front and rear of collective cell migration. Nat Rev Mol Cell Biol.

[5] Yamaguchi H, Condeelis J (2007). Regulation of the actin cytoskeleton in cancer cell migration and invasion. Biochim Biophys Acta.

[6] Wu H, Parsons JT (1993). Cortactin, an 80/85-kilodalton pp60Src substrate, is a filamentous actin-binding protein enriched in the cell cortex. J Cell Biol.

[7] Kruchten AE, Krueger EW, Wang Y, McNiven MA (2008). Distinct phospho-forms of cortactin differentially regulate actin polymerization and focal adhesions. Am J Physiol Cell Physiol.

[8] Weed SA, Parsons JT (2001). Cortactin: coupling membrane dynamics to cortical actin assembly. Oncogene.

[9] Weaver AM (2008). Cortactin in tumor invasiveness. Cancer Lett.

[10] MacGrath SM, Koleske AJ (2012). Cortactin in cell migration and cancer at a glance. J Cell Sci.

[11] Schuuring E, Verhoeven E, Mooi WJ, Michalides RJ (1992). Identification and cloning of two overexpressed genes, U21B31/PRAD1 and EMS1, within the amplified chromosome 11q13 region in human carcinomas. Oncogene.

[12] Hofman P, Butori C, Havet K, Hofman V, Selva E, Guevara N (2008). Prognostic significance of cortactin levels in head and neck squamous cell carcinoma: comparison with epidermal growth factor receptor status. Br J Cancer.

[13] Chuma M, Sakamoto M, Yasuda J, Fujii G, Nakanishi K, Tsuchiya A (2004). Overexpression of cortactin is involved in motility and metastasis of hepatocellular carcinoma. J Hepatol.

[14] Kim YN, Choi JE, Bae JS, Jang KY, Chung MJ, Moon WS (2012). Expression of cortactin and focal adhesion kinase in colorectal adenocarcinoma: correlation with clinicopathologic parameters and their prognostic implication. Korean J Pathol.

[15] Xu X-Z, Garcia MV, Li T, Khor L-Y, Gajapathy RS, Spittle C (2010). Cytoskeleton alterations in melanoma: aberrant expression of cortactin, an actin-binding adapter protein, correlates with melanocytic tumor progression. Mod Pathol..

[16] Patel AS, Schechter GL, Wasilenko WJ, Somers KD (1998). Overexpression of EMS1/cortactin in NIH3T3 fibroblasts causes increased cell motility and invasion in vitro. Oncogene.

[17] Huang C, Liu J, Haudenschild CC, Zhan X (1998). The role of tyrosine phosphorylation of cortactin in the locomotion of endothelial cells. J Biol Chem.

[18] Head JA, Jiang D, Li M, Zorn LJ, Schaefer EM, Parsons JT (2003). Cortactin tyrosine phosphorylation requires Rac1 activity and association with the cortical actin cytoskeleton. Mol Biol Cell.

[19] Okamura H, Resh MD (1995). p80/85 cortactin associates with the Src SH2 domain and colocalizes with v-Src in transformed cells. J Biol Chem.

[20] Tehrani S, Tomasevic N, Weed S, Sakowicz R, Cooper JA (2007). Src phosphorylation of cortactin enhances actin assembly. Proc Natl Acad Sci USA.

[21] Bowden ET, Onikoyi E, Slack R, Myoui A, Yoneda T, Yamada KM (2006). Co-localization of cortactin and phosphotyrosine identifies active invadopodia in human breast cancer cells. Exp Cell Res.

[22] Tsai W-C, Lin C-K, Lee H-S, Gao H-W, Nieh S, Chan D-C (2013). The correlation of cortactin and fascin-1 expression with clinicopathological parameters in pancreatic and ampulla of Vater adenocarcinoma. APMIS.

[23] Fantozzi I, Grall D, Cagnol S, Stanchi F, Sudaka A, Brunstein M-C (2008). Overexpression of cortactin in head and neck squamous cell carcinomas can be uncoupled from augmented EGF receptor expression. Acta Oncol.

[24] Artym VV, Zhang Y, Seillier-Moiseiwitsch F, Yamada KM, Mueller SC (2006). Dynamic interactions of cortactin and membrane type 1 matrix metalloproteinase at invadopodia: defining the stages of invadopodia formation and function. Cancer Res.

[25] Clark ES, Whigham AS, Yarbrough WG, Weaver AM (2007). Cortactin is an essential regulator of matrix metalloproteinase secretion and extracellular matrix degradation in invadopodia. Cancer Res.

[26] Nam S, Kim D, Cheng JQ, Zhang S, Lee J-H, Buettner R (2005). Action of the Src family kinase inhibitor, dasatinib (BMS-354825), on human prostate cancer cells. Cancer Res.

[27] Nagaraj NS, Smith JJ, Revetta F, Washington MK, Merchant NB (2010). Targeted inhibition of SRC kinase signaling attenuates pancreatic tumorigenesis. Mol Cancer Ther.

[28] Luebke-Wheeler JL, Nedredal G, Le Y, Amiot BP, Nyberg SL (2009). E-cadherin protects primary hepatocyte spheroids from cell death by a caspase-independent mechanism. Cell Transplant..

[29] Bryce NS, Clark ES, Leysath ML, Currie JD, Webb DJ, Weaver AM (2005). Cortactin promotes cell motility by enhancing lamellipodial persistence. Curr Biol..

[30] Hui R, Ball JR, Macmillan RD, Kenny FS, Prall OW, Campbell DH (1998). EMS1 gene expression in primary breast cancer: relationship to cyclin D1 and oestrogen receptor expression and patient survival. Oncogene.

[31] Zhao J, Wei J, Mialki R, Zou C, Mallampalli RK, Zhao Y (2012). Extracellular signal-regulated kinase (ERK) regulates cortactin ubiquitination and degradation in lung epithelial cells. J Biol Chem.

[32] Campbell DH, Sutherland RL, Daly RJ (1999). Signaling pathways and structural domains required for phosphorylation of EMS1/cortactin. Cancer Res.

[33] Mader CC, Oser M, Magalhaes MAO, Bravo-Cordero JJ, Condeelis J, Koleske AJ (2011). An EGFR-Src-Arg-cortactin pathway mediates functional maturation of invadopodia and breast cancer cell invasion. Cancer Res.

[34] Shields DJ, Murphy EA, Desgrosellier JS, Mielgo A, Lau SKM, Barnes LA (2011). Oncogenic Ras/Src cooperativity in pancreatic neoplasia. Oncogene.

[35] Crnogorac-Jurcevic T, Efthimiou E, Nielsen T, Loader J, Terris B, Stamp G (2002). Expression profiling of microdissected pancreatic adenocarcinomas. Oncogene.

[36] Li Y, Tondravi M, Liu J, Smith E, Haudenschild CC, Kaczmarek M (2001). Cortactin potentiates bone metastasis of breast cancer cells. Cancer Res.

[37] Huang J, Asawa T, Takato T, Sakai R (2003). Cooperative roles of Fyn and cortactin in cell migration of metastatic murine melanoma. J Biol Chem.

[38] Jia L, Uekita T, Sakai R (2008). Hyperphosphorylated cortactin in cancer cells plays an inhibitory role in cell motility. Mol Cancer Res.

[39] Miyazawa Y, Uekita T, Hiraoka N, Fujii S, Kosuge T, Kanai Y (2010). CUB domain-containing protein 1, a prognostic factor for human pancreatic cancers, promotes cell migration and extracellular matrix degradation. Cancer Res.

[40] Erami Z, Herrmann D, Warren SC, Nobis M, McGhee EJ, Lucas MC (2016). Intravital FRAP imaging using an E-cadherin-GFP mouse reveals disease- and drug-dependent dynamic regulation of cell-cell junctions in live tissue. Cell Rep.

[41] Canel M, Serrels A, Miller D, Timpson P, Serrels B, Frame MC (2010). Quantitative in vivo imaging of the effects of inhibiting integrin signaling via Src and FAK on cancer cell movement: effects on E-cadherin dynamics. Cancer Res.

[42] Magalhaes MAO, Larson DR, Mader CC, Bravo-Cordero JJ, Gil-Henn H, Oser M (2011). Cortactin phosphorylation regulates cell invasion through a pH-dependent pathway. J Cell Biol.

[43] Huang C, Ni Y, Wang T, Gao Y, Haudenschild CC, Zhan X (1997). Down-regulation of the filamentous actin cross-linking activity of cortactin by Src-mediated tyrosine phosphorylation. J Biol Chem.

[44] Li GH, Arora PD, Chen Y, McCulloch CA, Liu P (2012). Multifunctional roles of gelsolin in health and diseases. Med Res Rev.

[45] Thompson CC, Ashcroft FJ, Patel S, Saraga G, Vimalachandran D, Prime W (2007). Pancreatic cancer cells overexpress gelsolin family-capping proteins, which contribute to their cell motility. Gut.

[46] Tanaka H, Shirkoohi R, Nakagawa K, Qiao H, Fujita H, Okada F (2006). siRNA gelsolin knockdown induces epithelial-mesenchymal transition with a cadherin switch in human mammary epithelial cells. Int J Cancer.

[47] Lamouille S, Xu J, Derynck R (2014). Molecular mechanisms of epithelial–mesenchymal transition. Nat Rev Mol Cell Biol.

[48] Christiansen JJ, Rajasekaran AK (2006). Reassessing epithelial to mesenchymal transition as a prerequisite for carcinoma invasion and metastasis. Cancer Res.

[49] Sahai E (2005). Mechanisms of cancer cell invasion. Curr Opin Genet Dev.

[50] Gagliano N, Celesti G, Tacchini L, Pluchino S, Sforza C, Rasile M (2016). Epithelial-to-mesenchymal transition in pancreatic ductal adenocarcinoma: characterization in a 3D-cell culture model. World J Gastroenterol.

