# Supplementary material for: Overexpression and Tyr421-phosphorylation of cortactin is induced by three-dimensional spheroid culturing and contributes to migration and invasion of pancreatic ductal adenocarcinoma (PDAC) cells
Source: Cancer Cell Int. 2019 Mar 29;19:77. doi: 10.1186/s12935-019-0798-x (PMC6441202; doi:10.1186/s12935-019-0798-x)
Supplement: Supplementary file 1 — Additional file 1. Additional figures. [file 12935_2019_798_MOESM1_ESM.docx]

**Additional Figures**


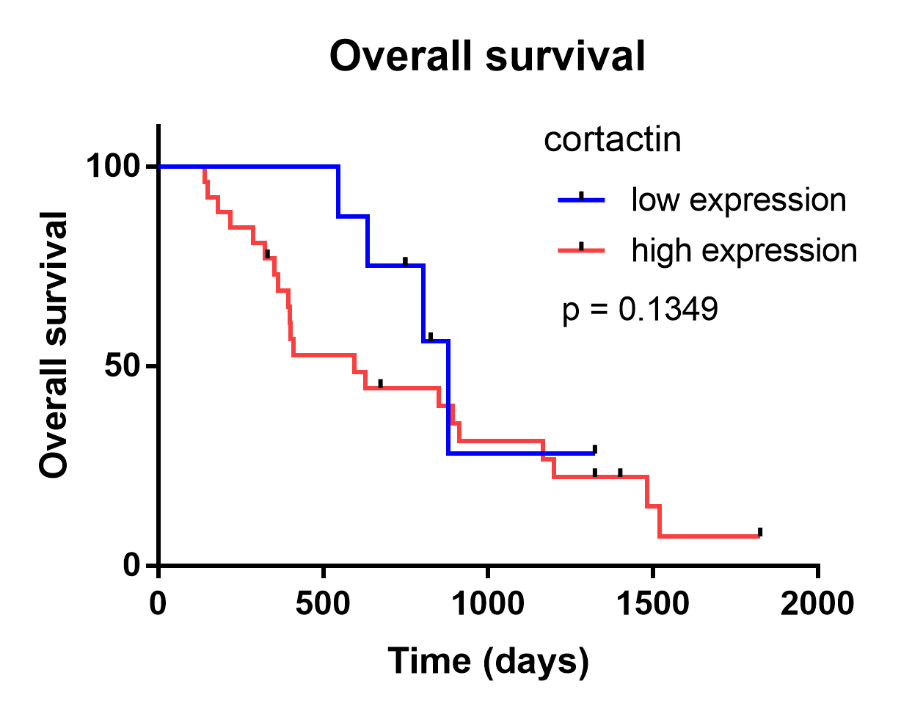


Additional file 1: Figure S1. Overall survival of PDAC patients (n=34) with low or high cortactin expression.


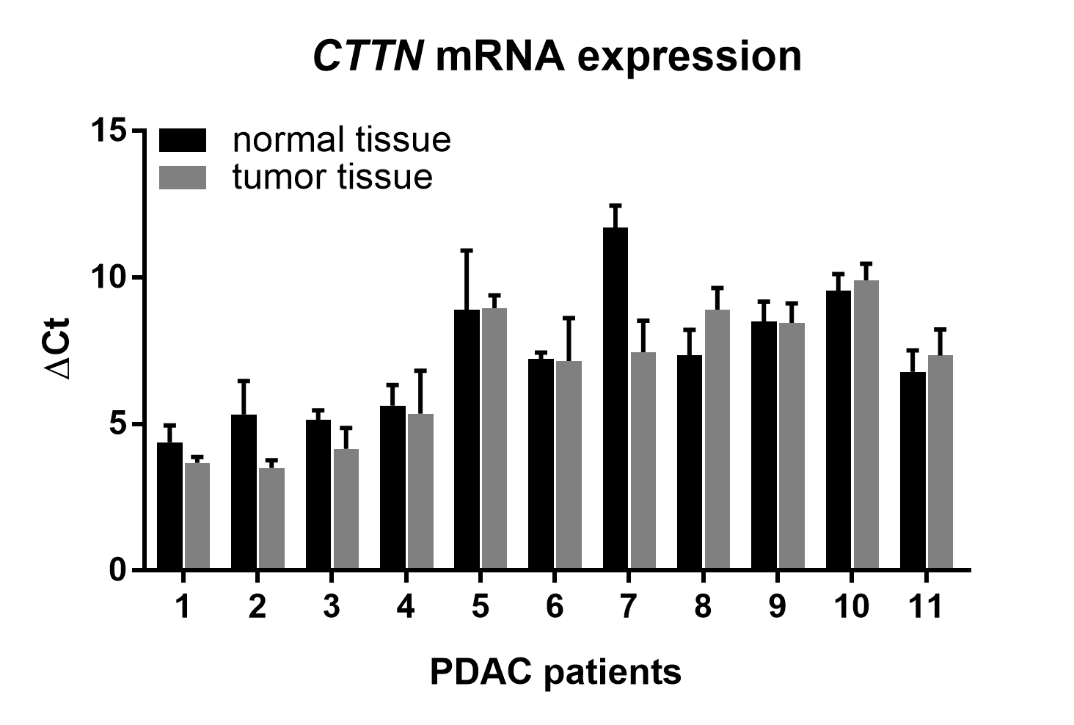


Additional file 1: Figure S2. Comparison of *CTTN* gene expression in non-neoplastic pancreatic tissue (normal tissue) and tumor tissue of pancreatic cancer patients. Expression levels are displayed as ΔCt values normalized to the housekeeping genes *GAPDH and RPLP0*.


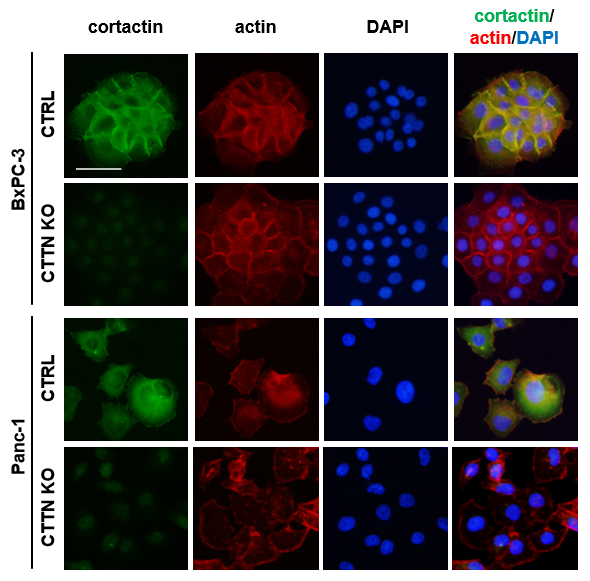


Additional file 1: Figure S3. Immunofluorescence of cortactin knockdown mediated by CRISPR/Cas9 technology revealed decreased cortactin expression in BxPC-3 and Panc‑1 CTTN KO cells compared to controls. Scale bar, 50 µm. CTRL, control.


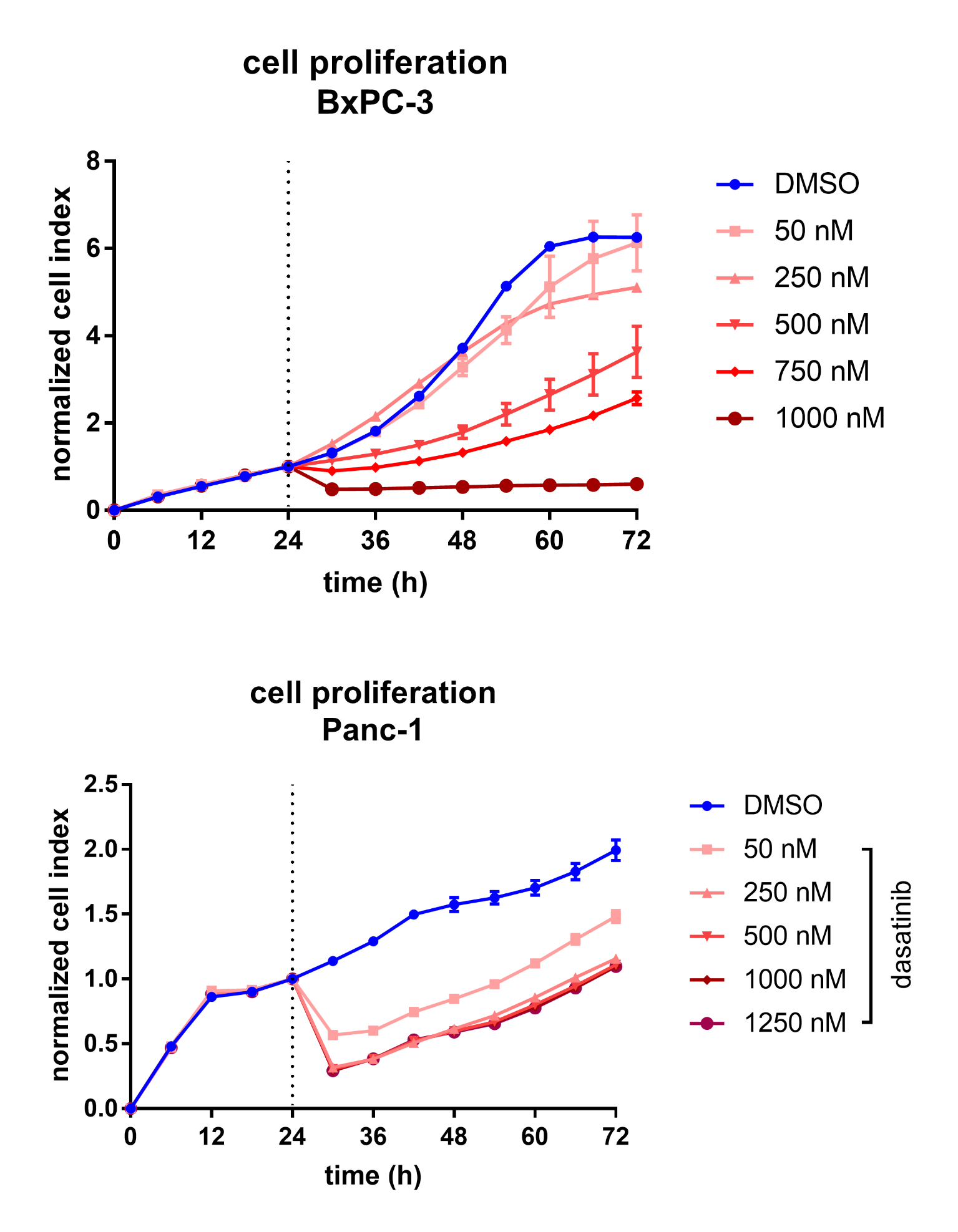


Additional file 1: Figure S4. Proliferation of pancreatic Panc-1 cells treated with dasatinib or DMSO as vehicle control. 10^4^ cells were seeded in duplicates into 16-well E-view plates and grown for 24 hours. Cells were treated with indicated concentrations of dasatinib or DMSO. In order to compare the effect of the inhibitor, the cell index curves were normalized to the last time point before the addition of the compound. Cell proliferation decreased directly after treatment, but after 5-6 hours the cells start to proliferate again with the same inclination rate as DMSO treated cells.


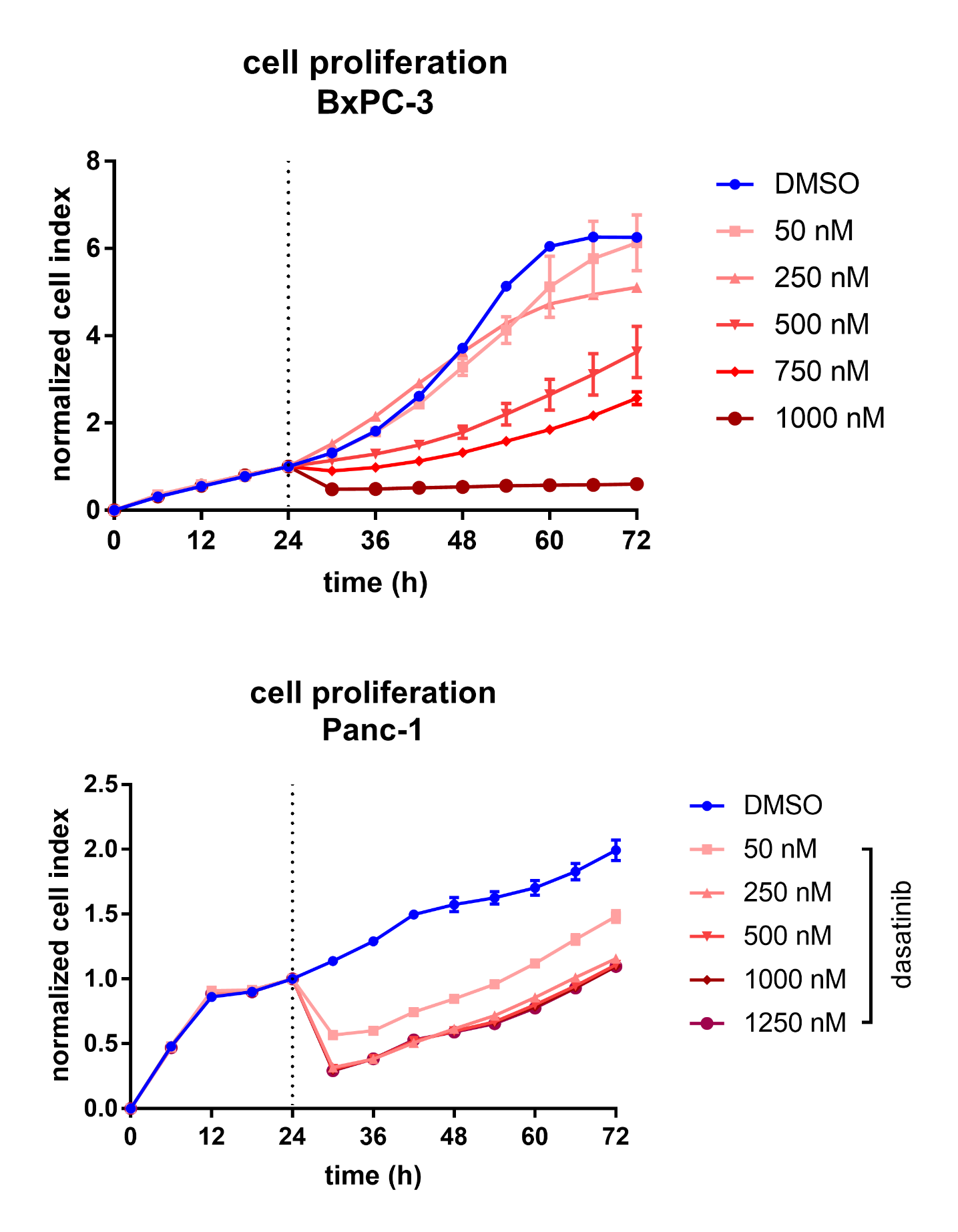


Additional file 1: Figure S5. Proliferation of pancreatic BxPC-3 cells treated with dasatinib or DMSO as vehicle control. 10^4^ cells were seeded in duplicates into 16-well E-view plates and grown for 24 hours. Cells were treated with indicated concentrations of dasatinib or DMSO.


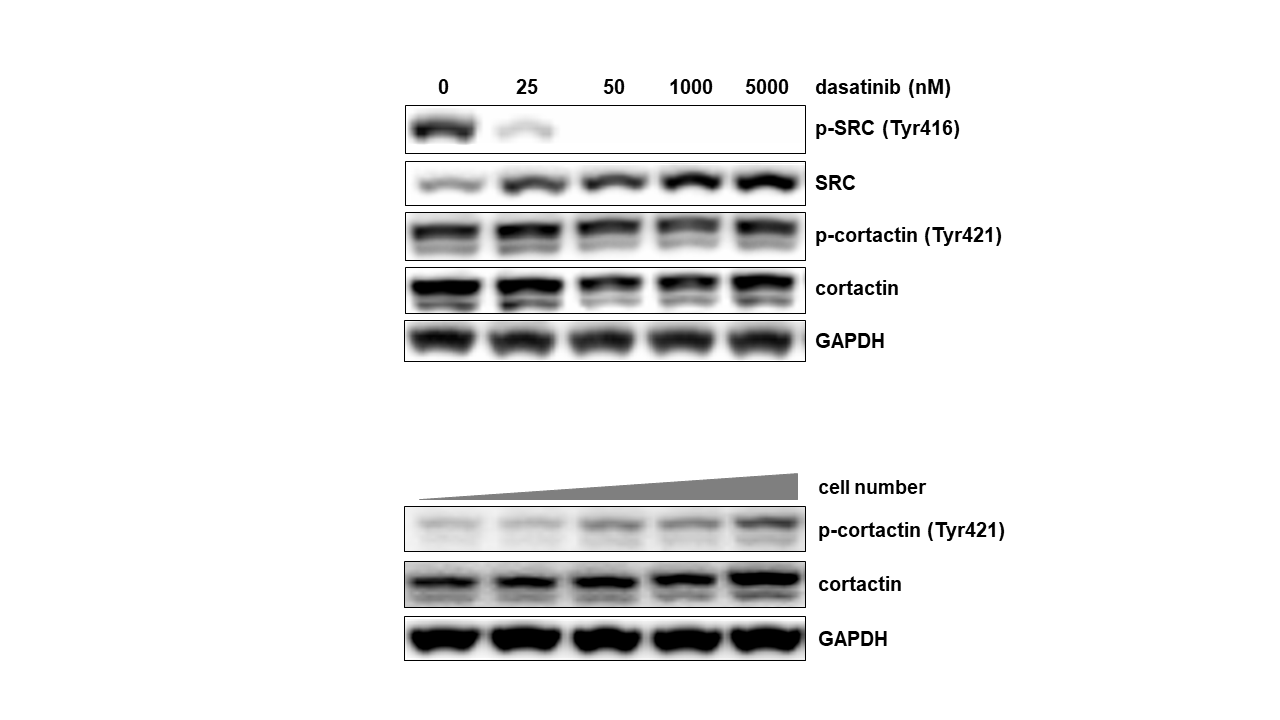


Additional file 1: Figure S6. Adherent cultured pancreatic Panc-1 cells were treated with the indicated dasatinib concentrations. An inhibitory effect of dasatinib on cortactin activation could not be detected after adherent cell culturing.


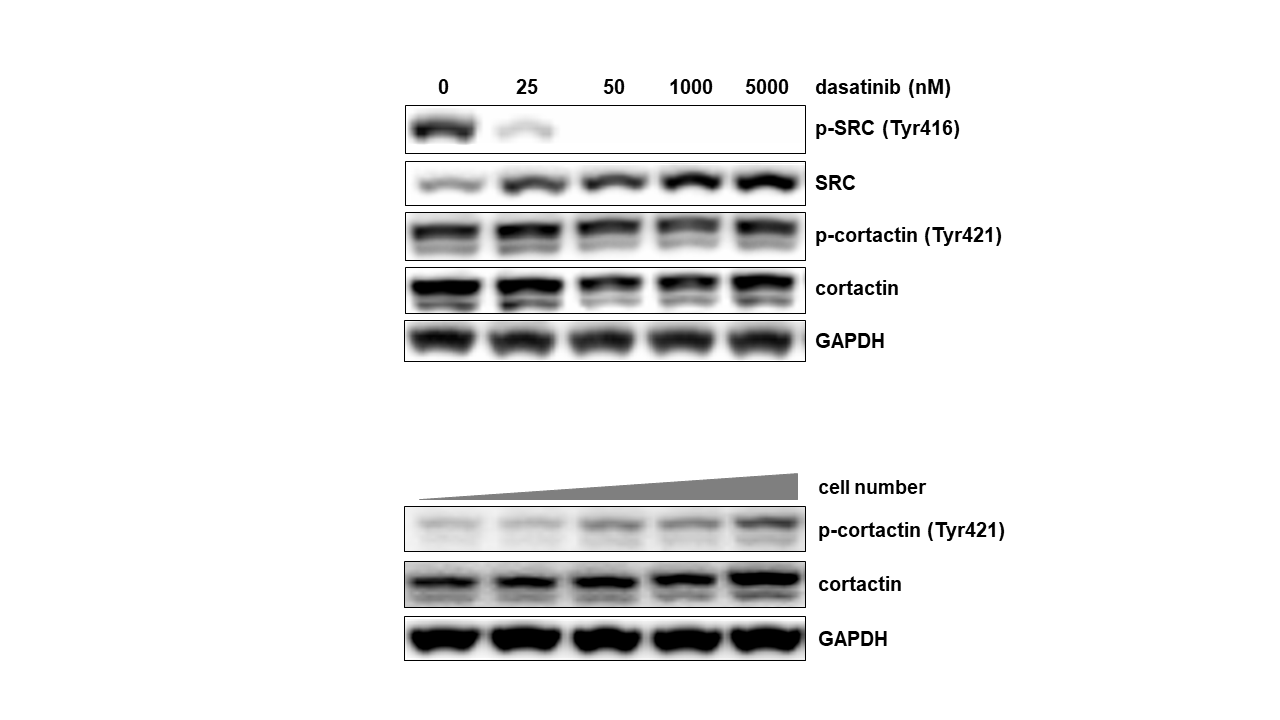


Additional file 1: Figure S7. Cortactin Tyr421-phosphorylation is enhanced with increasing cell confluency. Panc-1 cells were seeded in T25 cell culture flasks with increasing cell numbers (lane 1: 3.5x10^5^, lane 2: 5x10^5^, lane 3: 7.5x10^5^, lane 4: 1x10^6^, lane 6: 1.5x10^6^). Proteins were isolated when the cells seeded with the highest cell number were grown to a 100% confluent monolayer. GAPDH was included as loading control.
