# Supplementary material for: Overexpression and Tyr421-phosphorylation of cortactin is induced by three-dimensional spheroid culturing and contributes to migration and invasion of pancreatic ductal adenocarcinoma (PDAC) cells
Source: Cancer Cell Int. 2019 Mar 29;19:77. doi: 10.1186/s12935-019-0798-x (PMC6441202; doi:10.1186/s12935-019-0798-x)
Supplement: Supplementary file 2 — Additional file 2. Additional Materials and methods. [file 12935_2019_798_MOESM2_ESM.docx]

**Additional files to Overexpression and Tyr421-phosphorylation of cortactin is induced by three-dimensional spheroid culturing and contributes to migration and invasion of pancreatic ductal adenocarcinoma (PDAC) cells by Stock et al.**

**(Material and methods)**

**Gene expression analysis**

RNA from cryo-conserved tissues was extracted using peqGold Tri-Fast (Peqlab Biotechnology). RNA concentration was measured with a NanoDrop ND-1000 (Peqlab Biotechnology), and cDNA was synthesized from 1 µg RNA using the High-Capacity cDNA Reverse Transcription Kit (Applied Biosystems). Each diluted reverse transcription reaction (1:10) was combined with Power SYBR Green PCR Master Mix (Applied Biosystems) and the following primer pairs for cortactin: 5’-GGACCAAAAGCTTCCCATGG-3’ (sense) and 5’-CACCAAACTTGCCTCCGAAG-3’ (antisense); for GAPDH: 5’-acagtcagccgcatcttctt-3’ (sense) and 5’-gttaaaagcagccctggtga-3’ (antisense) (eurofins-MWG/Operon). qRT-PCR was performed using the StepOnePlus Real Time PCR system (Applied Biosystems).
